# Public Awareness and Knowledge Gaps in Osteoarthritis Prevention in the UAE: A Cross-Sectional Study on Risk Factors and Lifestyle Influences

**DOI:** 10.7759/cureus.77915

**Published:** 2025-01-24

**Authors:** Omar Hamodat, Rand Ameed, Abdulla Alzarooni, Abdul Rahman M Elmohamed, Deema Zainal, Jenan J Alkandari, Mohamed Eladl

**Affiliations:** 1 College of Medicine, University of Sharjah, Sharjah, ARE; 2 Department of Basic Medical Sciences, College of Medicine, University of Sharjah, Sharjah, ARE

**Keywords:** cross-sectional study, health education, knowledge gaps, osteoarthritis, preventive health, public awareness, risk factors, uae

## Abstract

Background

Osteoarthritis (OA) is a prevalent joint disorder affecting people globally, including in the UAE, where urbanization, sedentary lifestyles, and dietary habits have increased its occurrence. This study examines public awareness of OA and preventive measures in the UAE, aiming to identify knowledge gaps and emphasize the need for educational interventions to promote healthier choices and OA prevention.

Methods

A cross-sectional study was conducted with a self-administered online questionnaire distributed widely across internet platforms. The survey collected demographic information, factors influencing OA prevention knowledge, and participants’ awareness of OA prevention and associated risk factors. A total of 394 UAE residents participated. Data analysis was conducted using IBM SPSS Statistics for Windows, Version 29 (Released 2023; IBM Corp., Armonk, New York, United States).

Results

Among the 394 participants, 45.4% were aged 18-29, with nearly equal gender representation (51.3% male, 48.7% female). Most held undergraduate degrees (57.6%), and most (57.4%) were Arab non-Emiratis. Regarding OA awareness, 44.2% had low knowledge levels, while 21.8% showed high awareness. Nearly all respondents (95.7%) identified OA as joint-related, and 73.6% recognized weight management as preventive, though only 67% knew OA could affect multiple joints. Common misconceptions included beliefs that OA equally affects genders (27.9%) and that it’s caused by cold, damp weather (22.8%). Awareness of treatments like physiotherapy was moderate (66.8%), yet knowledge of advanced options like injections was low (38.8%). Significant correlations were found between OA awareness and factors like age, ethnicity, education, employment, and income.

Conclusion

Limited public awareness of OA and preventive measures exists in the UAE, underscoring the need for targeted public health education to address misconceptions and improve understanding.

## Introduction

Osteoarthritis (OA) is a progressive, degenerative joint disorder characterized by the breakdown of cartilage and surrounding tissues, leading to chronic pain, joint stiffness, and functional limitations [1,2]. As the most common form of arthritis worldwide, OA affects over 500 million people and is a leading cause of disability [3]. The burden of OA is profound, not only on the individuals experiencing it but also on healthcare systems that face increasing demands for effective management solutions and support services.

In the UAE, a sharp increase in OA prevalence is driven by lifestyle shifts, including urbanization, sedentary behaviors, and dietary changes, making disease prevention and management an urgent public health priority [4,5]. As people transition to more urbanized environments, they often adopt less physically active lifestyles and diets high in processed foods, which contribute to the risk factors associated with OA. Raising public awareness about OA is essential for prevention and early intervention. Informed populations are more likely to engage in preventive behaviors such as regular physical activity, weight management, and healthy dietary habits [6]. Physical activity, for instance, has been shown to strengthen muscles around the joints, reducing their load and potentially delaying OA onset. Similarly, weight management is crucial in lowering excess strain on weight-bearing joints, while balanced dietary habits can help mitigate inflammation [6,7].

Conversely, inadequate knowledge often results in delayed diagnosis, worsening symptoms, and increased healthcare burdens, all of which impact the quality of life [7,8]. A lack of awareness not only hampers early diagnosis but also affects self-management practices, leaving individuals ill-prepared to handle the condition’s progression. Enhancing public understanding of OA, especially around modifiable lifestyle factors, is thus critical to reducing its physical, social, and economic consequences [9]. Effective public health strategies that promote awareness and self-management have the potential to lessen the disease's impact by equipping individuals with tools to protect their joint health and manage symptoms early on.

In the UAE, urbanization and demographic trends contribute significantly to the rising rates of OA. Age, gender, and occupational influences further shape disease risk, with elderly populations and women particularly susceptible [10,11]. Age is a well-known risk factor for OA, as the wear and tear on joints accumulate over time. Gender disparities may also stem from both biological factors and occupational roles traditionally held by men and women. For instance, occupations involving repetitive joint movement or heavy physical labor can accelerate joint degeneration, increasing OA risk [10].

Effective prevention strategies including exercise, weight control, and dietary modifications have proven beneficial in lowering OA risk, especially when promoted through public health campaigns [12,13]. These interventions are often simple yet powerful, offering individuals clear, actionable steps to protect their joints and minimize risk. Public health campaigns that emphasize preventive measures, particularly highlighting the irreversible nature of OA and the importance of early intervention, can significantly encourage these behaviors, especially in communities where misconceptions about OA remain prevalent.

Despite increasing attention to OA, studies highlight persistent knowledge gaps in the UAE population, with common misconceptions about the disease’s causes, symptoms, and prevention [14,15]. Misunderstandings regarding OA’s origin, progression, and management options can lead individuals to ignore early warning signs or to believe that OA is an inevitable part of aging, leading them to neglect preventive care. These misunderstandings act as barriers to timely care and self-management, underscoring the need for educational programs that address these gaps with clear, actionable information [16]. Such programs would ideally cover not only basic OA education but also specific lifestyle-related prevention practices that are both accessible and relevant to diverse demographic groups in the UAE.

This study assesses public awareness of OA in the UAE, focusing on knowledge gaps in lifestyle-related prevention behaviors. By evaluating current awareness and highlighting areas for targeted health promotion, this research aims to empower individuals with preventive knowledge, improve quality of life, and alleviate the healthcare burden associated with OA in the UAE. The findings will inform strategies for public health intervention, contributing to a more comprehensive approach to managing OA within the community. Through effective education and outreach, this study seeks to foster a well-informed population that can make proactive choices to support joint health and minimize OA’s impact on individuals and society alike.

## Materials and methods

Study sample and setting

This cross-sectional, observational study was conducted in the United Arab Emirates from February 19 to March 7, 2024, using self-administered questionnaires. The study received approval from the Research Ethics Committee (REC) at the University of Sharjah (approval number: REC-24-01-24-01-5). Informed consent was obtained from all participants prior to their inclusion in the study. A sample size of 384 participants was required to accurately assess the level of knowledge regarding OA prevention methods.

Sample size

The estimated sample size was calculated using the formula: ss = (Z²pq)/c², where ss represents the sample size, (Z = 1.96), (p=0.5), (q = (1-p) = 0.5), and (c = 5 %) as the margin of sampling error. A total of 394 participants completed the questionnaire, with incomplete responses excluded from the analysis. Eligible participants were required to be mentally competent, residents of the United Arab Emirates, and willing to provide informed consent. The questionnaire was accessible online, with confidentiality and consent fully ensured.

Data collection

A team of medical students gathered the data by distributing the questionnaire to friends, family, and coworkers. It was also shared over several internet channels, including Telegram, emails, Twitter, and WhatsApp. Three sections comprised the self-administered questionnaire: an initial section collected demographic information such as age, gender, ethnicity, education level, and economic status; a subsequent section investigated factors impacting knowledge regarding OA prevention; and a third section comprised a 28-item questionnaire evaluating knowledge regarding osteoarthritis preventive measures and associated risk factors. The Alyami questionnaire was modified to meet the goals of this investigation [17]. Dr. Mohamed Eladl, the principal investigator, examined and approved every item.

Statistical analysis

The percentages representing the individuals' sociodemographic traits were derived using descriptive statistics. The metrics of central tendency and variability for quantitative variables were given as means and standard deviations (SD). Chi-square tests were used to assess the participants' knowledge of osteoarthritis and other qualitative characteristics. Knowledge levels were categorized based on participants' scores from the 28-item questionnaire. Scores were classified as 'adequate' for participants who achieved 60% or more, indicating sufficient awareness. Scores below this range were classified as 'insufficient.' For visualization purposes, these categories are labeled as 'pass' and 'fail'. A 95% confidence interval and a p-value of 0.05 or less were used to determine statistical significance. IBM SPSS Statistics for Windows, Version 29 (Released 2023; IBM Corp., Armonk, New York, United States) was used for all analyses.

## Results

A total of 394 participants completed the study questionnaire, with ages ranging from 18 to over 40 years. The largest age group comprised individuals aged 18-29 years (45.4%, n=179), followed by those aged 30-39 years (33.2%, n=131), and participants aged 40 and above (21.3%, n=84). Gender distribution was nearly balanced, with 51.3% male (n=202) and 48.7% female (n=192). Regarding educational attainment, 57.6% (n=227) held an undergraduate degree, 25.9% (n=102) had a high school diploma or lower, and 16.5% (n=65) possessed postgraduate qualifications.

The majority of participants identified as Arab non-Emiratis (57.4%, n=226), followed by Emiratis (22.6%, n=89), and non-Arabs (20.1%, n=79). Geographically, 50.3% (n=198) resided in Sharjah, while 39.8% (n=157) were from Dubai and Abu Dhabi, and 9.9% (n=39) were from the Northern Emirates. Income levels varied, with 37.3% (n=147) earning less than AED 5,000, while 4.6% (n=18) earning over AED 50,000. Employment status among participants was diverse: 53.8% (n=212) were employed, 28.9% (n=114) were students, and 17.3% (n=68) were unemployed.

Regarding physical characteristics, 40.6% (n=160) of participants had a normal BMI, 34.3% (n=135) were classified as overweight, and 21.1% (n=83) were categorized as obese. Knowledge about OA varied, with 44.2% (n=174) demonstrating low knowledge, 34% (n=134) exhibiting moderate knowledge, and 21.8% (n=86) displaying high knowledge. Notably, a vast majority (95.7%, n=377) correctly identified OA as a joint-related disease, and 51.8% (n=204) reported knowing someone diagnosed with OA (see Table 1).

**Table 1:** Personal data of study participants from the United Arab Emirates

Personal data	Number	%
Gender		
Male	202	51.3
Female	192	48.7
Age in years		
18-29	179	45.4
30-39	131	33.2
40 and above	84	21.3
Ethnicity		
Emirati	89	22.6
Arab (non-Emirati)	226	57.4
Non-Arab	79	20.1
Educational level		
High school diploma or less	102	25.9
Undergraduate	227	57.6
Postgraduate	65	16.5
City		
Northerner emirates	39	9.9
Sharjah	198	50.3
Dubai and Abu Dhabi	157	39.8
Income level		
Less than 5000	147	37.3
5000 – less than 15000	115	29.2
15000 – less than 50000	114	28.9
50000 and above	18	4.6
Employment status		
Student	114	28.9
Unemployed	68	17.3
Employed	212	53.8
BMI		
Underweight	16	4.1
Normal	160	40.6
Overweight	135	34.3
Obese	83	21.1
Exercise		
Low physical activity	212	53.8
Moderate physical activity	113	28.7
High physical activity	69	17.5
Knowledge		
Low knowledge	174	44.2
Moderate knowledge	134	34
High knowledge	86	21.8
Definition of Osteoarthritis		
It’s a disease that’s related to joints	377	95.7
It’s a disease that’s not related to joints	17	4.3
Do you know anyone with osteoarthritis?		
Yes	204	51.8
No	190	48.2

**Figure 1:**
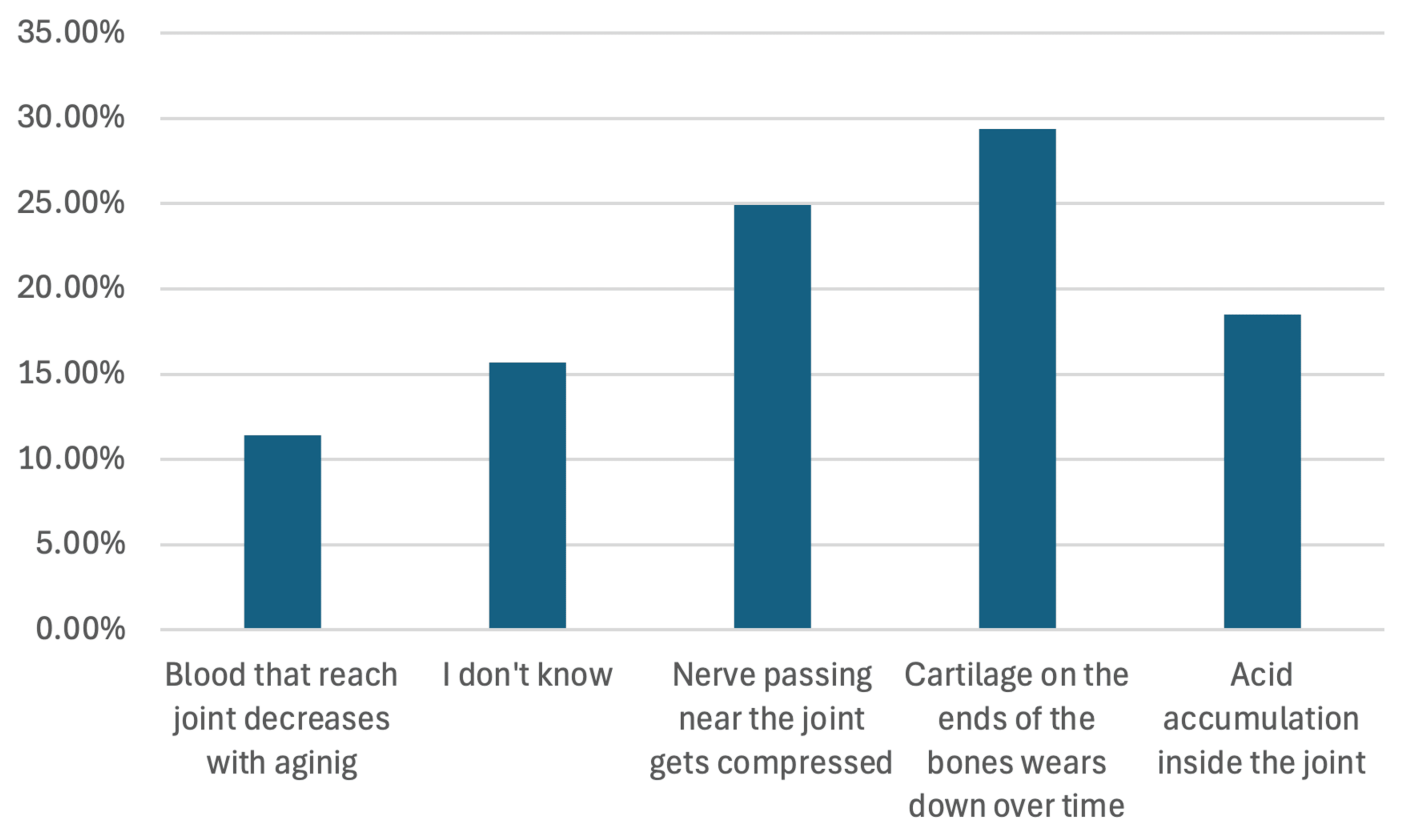
Participants' awareness regarding the mechanism of osteoarthritis development

Participants’ awareness of the mechanisms contributing to OA development varied significantly (see Figure 1). The most commonly identified cause was the wear and tear of cartilage (29.4%), followed by nerve compression near joints (24.9%). Misconceptions persisted, with 18.5% attributing OA to acid accumulation in the joints, and 15.7% expressing uncertainty about OA mechanisms.

When assessing risk factors, 74.6% (n=294) correctly identified aging as a major contributor, and 67% (n=264) recognized that OA can affect multiple joints. However, 27.9% (n=110) mistakenly believed that men and women are equally affected by OA, and 22.8% (n=90) inaccurately attributed the condition to cold, damp weather.

Awareness of preventive measures was generally encouraging, with 73.6% (n=290) acknowledging that maintaining a healthy weight can reduce OA risk, and 67.3% (n=265) recognizing the importance of managing joint stress through proper body mechanics. Nevertheless, only 38.6% (n=152) were aware of the need to avoid smoking as a preventive measure.

In terms of treatment options, 66.8% (n=263) recognized physiotherapy as a beneficial approach for managing OA symptoms, while 44.7% (n=176) acknowledged the role of NSAIDs (nonsteroidal anti-inflammatory drugs) in providing relief. Additionally, 59.9% (n=236) understood that exercises such as swimming are suitable for OA patients. However, knowledge regarding advanced treatments was less prevalent, with only 38.8% (n=153) aware of the benefits of intra-articular injections and 42.6% (n=168) recognizing joint replacement surgery as a definitive solution for alleviating OA symptoms (see Table 2).

**Table 2:** Community awareness about osteoarthritis and its related risk factors in the United Arab Emirates

Number	Statement	Yes	%	No	%	I don't know	%
1	Osteoarthritis is a chronic problem	252	64	53	13.5	89	22.6
2	Osteoarthritis is rare	37	9.4	270	68.5	87	22.1
3	Different joints can be affected by osteoarthritis	264	67	43	10.9	87	22.1
4	Osteoarthritis is caused by cold, damp weather	90	22.8	170	43.1	134	34
5	Osteoarthritis is developed microorganism (such as bacteria, virus, fungi...)	57	14.5	197	50	140	35.5
6	Pain is the only symptom of osteoarthritis	87	22.1	224	56.9	83	21.1
7	Stiffness is a symptom of osteoarthritis	234	59.4	47	11.9	113	28.7
8	Swelling is a sign of osteoarthritis	209	53	68	17.3	117	29.7
9	Osteoarthritis can lead to loss of joint movement	259	65.7	39	9.9	96	24.4
10	There are genetic factors that can predispose a person to osteoarthritis	188	47.7	78	19.8	128	32.5
11	Aging is a risk factor for osteoarthritis	294	74.6	30	7.6	70	17.8
12	Men and women are equally affected by osteoarthritis	110	27.9	164	41.6	120	30.5
13	Osteoarthritis is preventable	242	61.4	49	12.4	103	26.1
14	Maintaining a healthy weight can prevent osteoarthritis	290	73.6	40	10.2	64	16.2
15	Protecting joints from injuries can prevent osteoarthritis	232	58.9	65	16.5	97	24.6
16	Managing joint stress through proper body mechanics would help prevent osteoarthritis	265	67.3	42	10.7	87	22.1
17	Regular work setting can prevent osteoarthritis	221	56.1	58	14.7	115	29.2
18	Avoiding smoking would contribute to osteoarthritis prevention	152	38.6	107	27.2	135	34.3
19	Regular health check-ups can play a role in the prevention of osteoarthritis	278	70.6	39	9.9	77	19.5
20	Physical examination and x-ray are used to diagnose osteoarthritis	263	66.8	32	8.1	99	25.1
21	Blood tests are used to diagnose osteoarthritis	123	31.2	117	29.7	154	39.1
22	NSAIDs (pain killers) can improve osteoarthritis symptoms	176	44.7	102	25.9	116	29.4
23	All physical activities prevent osteoarthritis	106	26.9	169	42.9	119	30.2
24	Some forms of exercise like swimming are suitable for people with osteoarthritis	236	59.9	41	10.4	117	29.7
25	Acid-free diets are a proven treatment for osteoarthritis	122	31	72	18.3	200	50.8
26	Physiotherapy can cause a great improvement in the symptoms of osteoarthritis	263	66.8	34	8.6	97	24.6
27	Intra-articular injection by stem cell or hyaluronic acid is an effective modality for curing osteoarthritis	153	38.8	70	17.8	171	43.4
28	A joint replacement surgery will be the ultimate option to relieve the symptoms of osteoarthritis	168	42.6	77	19.5	149	37.8

**Figure 2:**
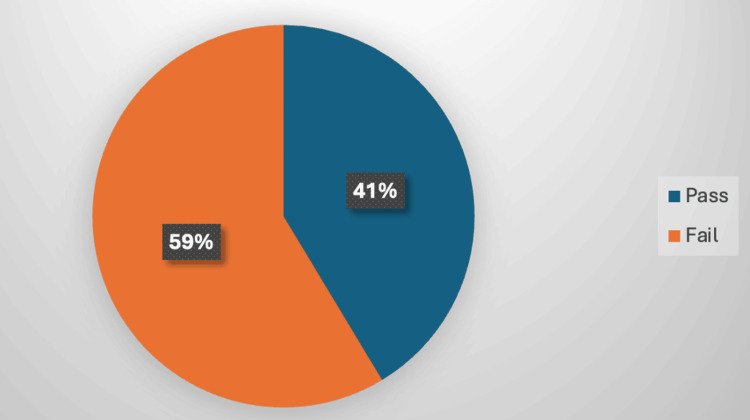
Overall community awareness level about osteoarthritis and its related risk factors in the United Arab Emirates

In summary, only 163 participants (41.3%) demonstrated an adequate level of knowledge regarding preventive measures for OA, while 231 participants (58.7%) exhibited insufficient knowledge (see Figure 2).

As shown in Table 3, age significantly influenced awareness levels, with participants aged 30-39 demonstrating the highest awareness (51.9%, p=0.001) compared to those aged 18-29 (30.2%). Ethnicity also impacted awareness, as non-Arabs exhibited higher levels of knowledge (59.5%, p=0.001) than Arabs (42.9%). Furthermore, higher educational levels correlated with greater awareness, particularly among those holding postgraduate degrees (61.5%, p=0.001).

**Table 3:** Factors associated with participants’ awareness regarding osteoarthritis

Factors	Awareness level				p-value
	Pass		Fail		
	Number	%	Number	%	
Gender					0.465
Male	80	39.6	122	60.4	
Female	83	43.2	109	56.8	
Age in years					0.001
18-29	54	30.2	125	69.8	
30-39	68	51.9	63	48.1	
40 and above	41	48.8	43	51.2	
Ethnicity					0.001
Emirati	19	21.3	70	78.7	
Arab (non-Emirati)	97	42.9	129	57.1	
Non- Arab	47	59.5	32	40.5	
Educational level					0.001
High school diploma or less	33	32.4	69	67.6	
Undergraduate	90	39.6	137	60.4	
Postgraduate	40	61.5	25	38.5	
City					0.001
Northerner emirates	14	35.9	25	64.1	
Sharjah	66	33.3	132	66.7	
Dubai and Abu Dhabi	83	52.9	74	47.1	
Income level					0.004
Less than 5000	49	33.3	98	66.7	
5000 – less than 15000	43	37.4	72	62.6	
15000 – less than 50000	61	53.5	53	46.5	
50000 and above	10	55.6	8	44.4	
Employment status					0.016
Student	35	30.7	79	69.3	
Unemployed	28	41.2	40	58.8	
Employed	100	47.2	112	52.8	
BMI					0.164
Underweight	4	25	12	75	
Normal	61	38.1	99	61.9	
Overweight	65	48.1	70	51.9	
Obese	33	39.8	50	60.2	
Exercise					0.284
Low physical activity	80	37.7	132	62.3	
Moderate physical activity	52	46	61	54	
High physical activity	31	44.9	38	55.1	
Knowledge					0.001
Low knowledge	36	20.7	138	79.3	
Moderate knowledge	65	48.5	69	51.5	
High knowledge	62	72.1	24	27.9	
Definition of Osteoarthritis					0.001
It’s a disease that’s related to joints	163	43.2	214	56.8	
It’s a disease that’s not related to joints	0	0	17	100	
Do you know anyone with osteoarthritis?					0.001
Yes	121	59.3	83	40.7	
No	42	22.1	148	77.9	
Source of information					0.001
No source	7	10.9	57	89.1	
Personal experience	20	41.7	28	58.3	
Relatives and friends	49	47.1	55	52.9	
Internet and social media	18	30	42	70	
Healthcare institutions and their staff	21	63.6	12	36.4	
School/University	45	60.8	29	39.2	
Mass media	3	27.3	8	72.7	

Geographically, respondents from Dubai and Abu Dhabi reported significantly greater awareness (52.9%) than those from Sharjah (33.3%) and the Northern Emirates (35.9%) (p=0.001). Income levels were also linked to awareness, with participants earning AED 50,000 and above showing the highest levels (55.6%, p=0.004). Employment status significantly influenced awareness, as employed participants had higher levels (47.2%, p=0.016) compared to students (30.7%). Notably, BMI and exercise levels did not show significant correlations with awareness (p=0.164 and p=0.284, respectively).

Knowledge levels were closely associated with awareness: 72.1% of participants with high knowledge also exhibited high awareness, in stark contrast to only 20.7% of those with low knowledge (p=0.001). Additionally, 43.2% of individuals who recognized OA as a joint-related disease demonstrated significantly better awareness than those who did not (0%) (p=0.001). Awareness was also notably higher among participants who knew someone with OA (59.3%) compared to those who did not (22.1%) (p=0.001).

**Figure 3:**
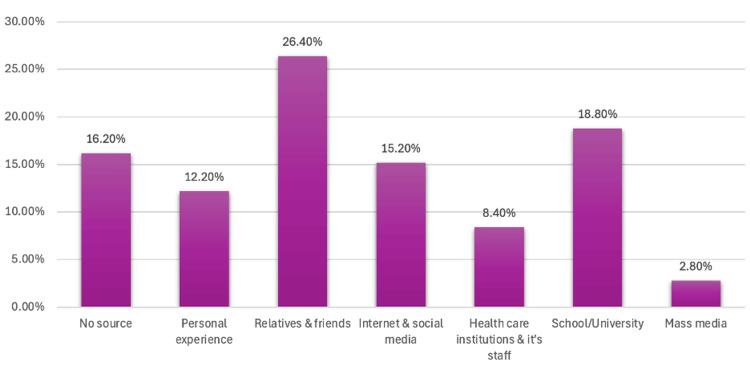
Sources of information about osteoarthritis and its related risk factors in the United Arab Emirates

Regarding sources of knowledge, Figure 3 illustrates that relatives and friends were the most common sources (26.4%), followed by educational institutions (18.8%) and the internet/social media (15.2%). Only 8.4% of participants received their information from healthcare institutions and staff, while 16.2% had no identifiable source of information. Participants who obtained their knowledge from healthcare institutions and educational sources exhibited significantly higher awareness levels (p=0.001), whereas those with no identifiable source of information displayed the lowest awareness (10.9%).

## Discussion

OA remains the most common musculoskeletal disorder globally, representing a pressing health issue due to its high prevalence and impact on quality of life. This cross-sectional study provides insight into the awareness levels regarding OA preventive measures among adults in the UAE, revealing both encouraging findings and critical knowledge gaps. The sample primarily consisted of younger adults aged 18 to 29, most of whom held an undergraduate degree, which reflects the demographic reached through an online self-administered questionnaire. These findings are consistent with a similar study conducted in Jeddah, Saudi Arabia, where younger, educated respondents also dominated the sample [17].

Among the 394 participants, only 41.3% (n=163) demonstrated an adequate knowledge level, leaving a significant portion of the population with insufficient awareness. While there was high recognition of OA as a joint-related disease (95.7%), understanding of its underlying mechanisms was inconsistent, with only 29.4% correctly identifying cartilage wear and tear as a primary cause. This underlines the need for educational initiatives that clarify OA’s pathophysiology and promote accurate knowledge among the UAE population.

Age was found to be a significant factor in OA awareness, with participants aged 30-39 exhibiting the highest awareness levels. This contrasts with a study from the Aseer region, where older adults (50+) demonstrated greater OA knowledge [18]. Additionally, education played a critical role, as participants with postgraduate qualifications showed significantly higher knowledge levels, suggesting that awareness efforts may be more impactful when tailored to different educational backgrounds.

Geographical variations were also evident, with respondents from Dubai and Abu Dhabi reporting higher awareness (52.9%) compared to those from Sharjah (33.3%) and the Northern Emirates (35.9%). This differs from a study in Sudair, Saudi Arabia, which found no correlation between knowledge levels and location, indicating that awareness may be influenced by the accessibility of health information and resources within UAE’s urban centers [19]. Furthermore, non-Arab participants demonstrated higher awareness than Arabs (59.5% vs. 42.9%), suggesting that cultural background may play a role in OA knowledge, with potential implications for how health education campaigns are crafted and delivered.

A personal connection to OA was a powerful influence on awareness levels, as participants who knew someone with OA showed significantly higher awareness (59.3%) compared to those without such connections (22.1%). This finding aligns with research from Malaysia, where personal experiences with OA were linked to better knowledge [20]. The study also highlights the importance of healthcare and educational institutions as information sources, with participants who gained knowledge through these channels showing significantly higher awareness than those without a clear source of information.

The study examined participants’ knowledge of OA preventive measures, with 73.6% understanding that maintaining a healthy weight is beneficial and 67.3% acknowledging the importance of joint stress management. However, awareness of smoking cessation as a preventive measure was notably low (38.6%), despite evidence linking smoking to OA progression. The significant number of participants (34.3%) who responded with “I Don’t Know” to prevention-related questions underscores a critical knowledge gap in understanding lifestyle factors that contribute to OA.

Awareness of treatment options was also variable. Physiotherapy was recognized by 66.8% as beneficial, and exercises like swimming were acknowledged by 59.9% of participants. This reflects a strong understanding of conservative management approaches, yet knowledge of advanced treatments, such as intra-articular injections (38.8%) and joint replacement surgery (42.6%), was limited. This disparity suggests that while basic treatment options are understood, there is insufficient awareness about more advanced interventions that could alleviate severe OA symptoms. Limited understanding of these options could delay timely and appropriate interventions, potentially leading to avoidable suffering and reduced quality of life for individuals with advanced OA.

The findings reveal a substantial knowledge gap regarding OA prevention in the UAE. Compared to countries such as the United States and Canada, where awareness levels are higher due to public health campaigns by organizations like the Arthritis Foundation and the Canadian Orthopedic Association, the UAE has room for growth in OA education. Increased public health campaigns, fostering open communication with healthcare professionals, and implementing educational programs are recommended strategies for bridging this knowledge gap. Social media, widely used in the UAE, offers an effective platform for awareness campaigns, providing an accessible channel for distributing information. Enhanced awareness can lead to healthier lifestyle choices, earlier OA detection, and ultimately better health outcomes for those at risk or already living with the condition.

In conclusion, the study underscores the need for comprehensive public health strategies that address both knowledge gaps and misconceptions about OA in the UAE. By targeting specific demographics and utilizing diverse communication channels, these strategies can foster a well-informed population capable of making proactive lifestyle decisions to manage and prevent OA. The findings from this study can guide future health promotion efforts, contributing to a healthier society with reduced OA prevalence and associated healthcare burdens.

Limitations

This study’s findings may have limited generalizability due to the inherent constraints of an online self-administered questionnaire, which restricts participation to literate individuals, who have internet access and are interested in participating. This approach likely contributed to the underrepresentation of older adults, a demographic at higher risk for OA. Additionally, the sample skewed younger, with 45.4% of participants aged between 18 and 29 years, indicating a potential selection bias.

## Conclusions

The study revealed a general lack of knowledge about OA among participants, with several misconceptions regarding its causes, risk factors, and management. These findings underscore the importance of increasing public awareness about OA and suggest that targeted educational initiatives may be beneficial. Further research across the region is essential to comprehensively assess public understanding of OA and inform future health interventions.

## Appendices

Questionnaire

https://drive.google.com/drive/u/0/folders/1WznNh48m2qXBs_-BBq4FCa0heqFqK1aP
